# Prognostic analysis of lung squamous cell carcinoma patients with second primary malignancies: a SEER database study

**DOI:** 10.3389/fonc.2024.1294383

**Published:** 2024-02-20

**Authors:** Weiqing Han, Silin Wang, Lang Su, Jianjun Xu, Yiping Wei

**Affiliations:** ^1^ Department of Cardiothoracic Surgery, The Second Affiliated Hospital, Jiangxi Medical College, Nanchang University, Nanchang, Jiangxi, China; ^2^ Department of Thoracic Surgery, The Second Affiliated Hospital, Jiangxi Medical College, Nanchang University, Nanchang, Jiangxi, China

**Keywords:** lung squamous cell carcinoma, SEER, second primary malignancy, survival time, NSCLC

## Abstract

**Background:**

As lung squamous cell carcinoma (LUSC) patients are at increased risk of developing a second primary cancer, this complicates the patient’s condition and thus makes prognostic assessment more difficult, posing a significant prognostic challenge for clinicians. Our goal was to assess the prognosis of LUSC patients with a second primary tumor, and provide insights into appropriate therapy and monitoring strategies.

**Methods:**

Data was obtained for LUSC patients from the Surveillance, Epidemiology, and End Results (SEER) database. The LUSC patients were divided into three groups (LS-SPM, OT-LUSC and LUSC-only). Univariate and stratified analyses were performed for the baseline and clinical characteristics of the participants. Multiple regression and Kaplan-Meier survival analyses were also performed, followed by a final life table analysis.

**Results:**

In our sample of 101,626 patients, the HR for OS in the LS-SPM group was 0.40 in univariate analysis. Kaplan-Meier survival curves showed that LS-SPM patients had considerably longer lifespans compared to the other groups. The LS-SPM patients had median and mean survival times of 64 months and 89.11 months. Unadjusted and adjusted multiple regression analyses showed that LS-SPM patients had a superior survival compared to LUSC-only and OT-LUSC groups.

**Conclusion:**

LS-SPM patients have a good prognosis with aggressive therapy and immune monitoring. The present study offers novel insights into the pathophysiological causes and treatments for LS-SPM.

## 1 Introduction

Lung cancer is the most common cancer worldwide, and has the highest fatality rate among cancers (1). Non-small cell lung cancer (NSCLC) constitutes the majority of lung cancers. Lung squamous cell carcinoma (LUSC), the second most common NSCLC subgroup, accounts for 20–30% of pulmonary cancers (2, 3). LUSC patients had a dismal 5-year survival rate, even with surgery and other treatments (4). The average duration of survival in cancer patients has increased significantly in recent years because of advancements in surgical methods and systemic therapies (5). Second primary malignancies (SPMs) in cancer survivors are becoming increasingly common in clinical practice (6). This presents significant difficulties in deciding the therapeutic options and assessing patient prognosis, so further exploratory studies are needed.

SPM is a tumor independent of and biologically distinct from the original primary tumor (7). The mechanism of SPM development remains unclear (8). Several studies have demonstrated that radiation therapy used to treat cancers may be responsible for the emergence of SPMs (9–11). Vogt et al. (12) reported that several distinctive factors, including cancer susceptibility syndromes, tumor characteristics, environmental exposure, and long-term treatment side-effects, may make cancer survivors more susceptible to SPMs. It was previously believed that cancer patients with SPMs had a poor prognosis. Consequently, harsher treatment modalities were discontinued (13). However, previous studies had several drawbacks, including obsolete data and samples that are not representative of the LUSC population, and their findings are debatable (14–16).

It is difficult to accurately guide treatment planning and prognosis in the absence of studies regarding the prognosis and survival of patients with LUSC and SPM (LS-SPM). This significantly reduces the willingness of patients and their families to opt for active treatment. There is growing concern for the prognosis of LS-SPM patients, and this requires further research. In the present study, we examined and combined the updates on LUSCs published in the SEER database in November 2021. In order to assess the impact on survival and progression of SPMs in LUSC patients, we analyzed the genuine survival rates of LS-SPM patients using the most recent data. This study may clarify the prognosis of LS-SPM patients, promote the development of effective treatment modalities, and highlight the importance of new discoveries in this field.

## 2 Methods

### 2.1 Data source

In November 2021, data was collected from the SEER program of the National Cancer Institute, including eight registries from 1975 to 2019. About 27.8% of the American demographic is included in the SEER database, which routinely gathers retrospective clinical data, including patient demographics, original tumor site, diagnostic stage, partial immunohistochemistry, and survival status. Our study focused on LUSCs, a type of NSCLCs. There were no missing values in the data for exposure and independent variables. We selected 19 entries, including patient ID, age (< 75 years or ≥ 75 years), ICD-O-3 Hist/behave, race (White, Black, or other), sex, grade, primary site, historic stage, positive regional nodes, laterality, marital status, radiation, surgery, chemotherapy, year of diagnosis, duration in months from diagnosis to treatment, status, survival months, and sequence number.

### 2.2 Data processing

**Figure 1:**
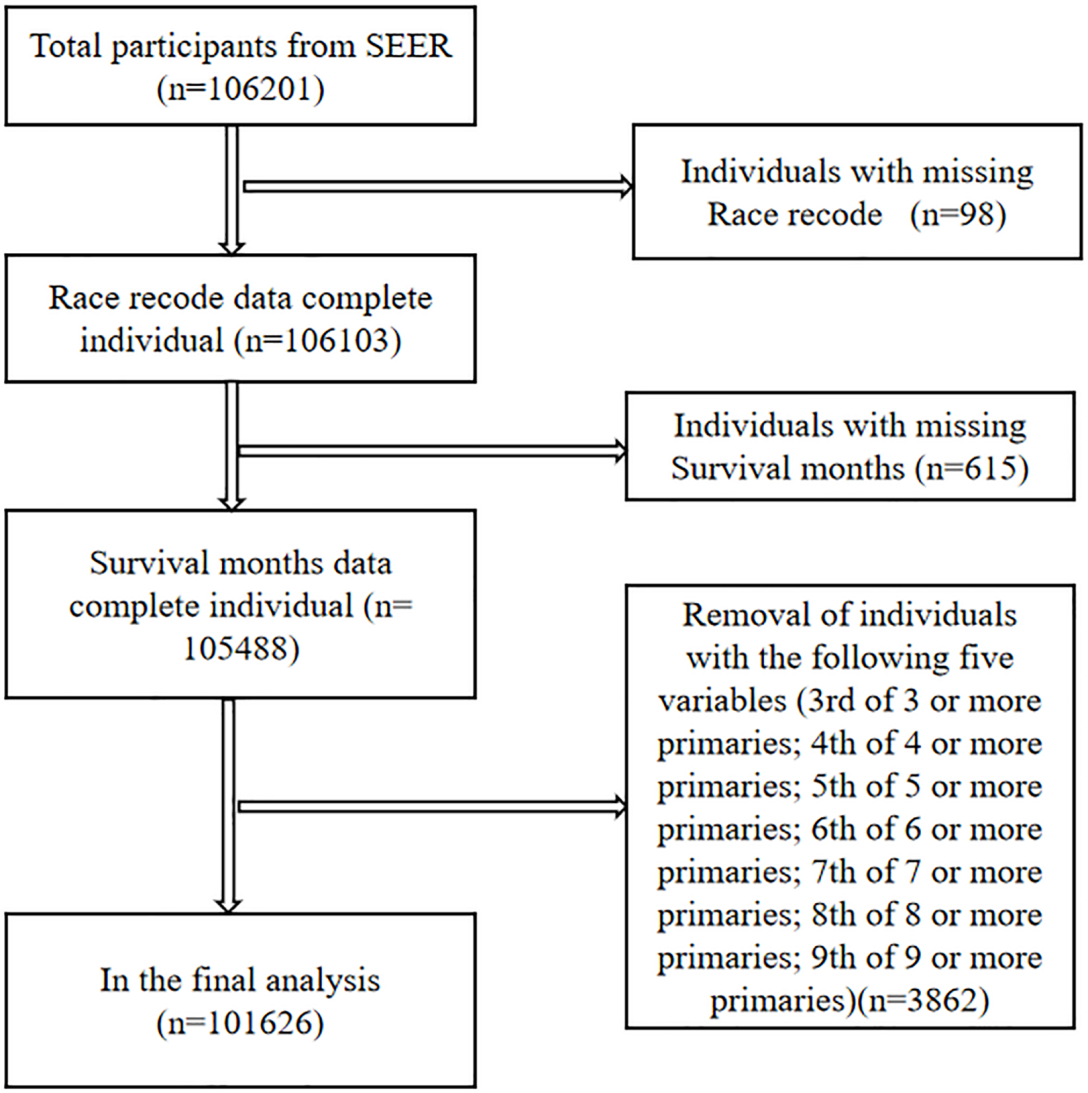
Data screening chart with LUSC patients.

We screened 106,201 pathologically diagnosed LUSC cases from the lung cancer section of the SEER database. Ninety-eight cases with missing race and 615 cases with missing survival months records were excluded. To make the data more robust, five items with the lowest number of cases in the sequence number record, for a total of 3862 cases, were removed, including 3rd of the three or more primaries, 4th of the four or more primaries, 5th of the five or more primaries, and so on. Finally, 101,626 LUSC patients were included (**Figure 1**). We combined and grouped individual entries in order to succinctly summarize the findings. We defined LUSC secondary to other primary malignancies (1^st^ of two or more primaries) as LS-SPM, other primary cancer secondary to LUSC (2^nd^ of two or more primaries) as OT-LUSC and patients with LUSC only as LUSC-only.

### 2.3 Statistical analysis

This study relied on the dataset from the SEER database and statistically screened the data through SPSS Statistics v.25.0. Statistical analyses were performed using Empower RCH software to screen the dataset, and baseline, univariate Cox proportional risk analysis, and stratified analysis tables were generated in order to comprehensively explore the effect of combined second primary tumor on the prognosis of patients with lung squamous cell carcinoma. We also performed multiple regression analysis using Empower RCH software. Even after accounting for confounding factors such as age, gender, stage, and treatment modality, this difference still showed significance when analyzed by three different models, further ensuring a comprehensive assessment of the impact of combined tumors. In addition, Kaplan-Meier survival curves for patients with different classifications of squamous lung cancer were plotted using R v.3.6.3 software for comparing survival rates. Also, analysis of other covariates was performed in GraphPad Prism 9 software to generate Kaplan-Meier survival curves to deepen the understanding of the impact of other covariates on survival. For a more comprehensive assessment of patient prognosis, we used SPSS to create a 3- and 5-year survival table and a life table analysis table for LS-SPM patients to more visually observe the prognosis. Through these statistical analyses and data visualization tools, we comprehensively revealed the prognostic characteristics of LS-SPM patients in multiple dimensions, which provided a strong scientific basis for clinical decision-making and further research.

## 3 Results

### 3.1 Baseline participant characteristics

Based on the inclusion criteria, 101,626 participants were screened from the SEER database, of whom 70892 (69.76%) were male, 30684 (30.19%) were aged > 75 years, and 86698 (85.31%) were white. The proportion of well- and moderately-differentiated LUSCs was 27473 (27.03%), with 82605 (81.28%) of the primary sites in lung lobes and 22150 (21.80%) distant metastases. Radiation therapy, surgery, and chemotherapy were performed in 53359 (52.51%), 29641 (29.17%), and 25820 (25.41%) of the cases, respectively. People who had a partner and unchecked lymph nodes were present in 58542 (57.61%) and 78105 (76.86%) cases, respectively. Treatment was initiated within 1 month of the diagnosis in 33322 (32.79%) of the cases. The remaining baseline characteristics are detailed in **Table 1**.

**Table 1:** Baseline characteristics of LUSC patients (N = 101,626).

Sequence number	N (%)	Single primary	1^st^ of two or more primaries	2^nd^ of two or more primaries	P-value
Sex					<0.001
Male	70892 (69.76%)	70.9	70.6	64.8	
Female	30734 (30.24%)	29.1	29.4	35.2	
Age					<0.001
< 75 years	70942 (69.81%)	71.5	78.3	59.5	
≥ 75 years	30684 (30.19%)	28.5	21.7	40.5	
Race					<0.001
White	86698 (85.31%)	84.7	86.1	87.5	
Black	8352 (8.22%)	8.4	8.2	7.5	
Others^(a)^	6576 (6.47%)	6.9	5.7	5	
Grade					<0.001
Grade 1^(b)^	27473 (27.03%)	25.8	36.3	28.6	
Grade 2^(c)^	38032 (37.42%)	37.9	40.2	34.3	
Unknown	36121 (35.54%)	36.3	23.5	37.1	
Primary site					<0.001
Main bronchus	6060 (5.96%)	6.5	3.6	4.5	
Lung lobe	82605 (81.28%)	79.6	90.2	84.7	
Other^(d)^	12961 (12.75%)	13.8	6.2	10.8	
Historic stage					<0.001
Localized	14564 (14.33%)	11.2	30.2	21.3	
Regional	22353 (22.00%)	21.4	25.2	23.3	
Distant	22150 (21.80%)	23.3	7.9	20.8	
Unknown	42559 (41.88%)	44.1	36.7	34.5	
Regional nodes					<0.001
Not examined	78105 (76.86%)	79.6	55.3	73.7	
Negative	13622 (13.40%)	10.8	33.1	16.8	
Positive	9899 (9.74%)	9.6	11.6	9.5	
Laterality					<0.001
Right	54861 (53.98%)	54.1	52.4	54	
Left	43388 (42.69%)	42.2	46.5	43.4	
Other^(e)^	3377 (3.32%)	3.7	1.1	2.7	
Marital status					<0.001
Partnered^(f)^	58542 (57.61%)	57.6	62	56	
Alone^(g)^	39782 (39.15%)	39.3	35.3	40.1	
Unknown	3302 (3.25%)	3.2	2.7	3.9	
Radiation					<0.001
Yes	53359 (52.51%)	55.2	35.7	47.7	
No	968 (0.95%)	1	0.5	0.9	
Unknown	47299 (46.54%)	43.8	63.8	51.5	
Surgery					<0.001
Yes	29641 (29.17%)	25.7	63.6	30.4	
No	67453 (66.37%)	69.2	34.8	66.7	
Unknown	4532 (4.46%)	5.1	1.6	2.9	
Chemotherapy					<0.001
Yes	25820 (25.41%)	25.8	21.7	25	
No	75806 (74.59%)	74.2	78.3	75	
Year of diagnosis					<0.001
1975–1989	34776 (34.22%)	37.3	31.7	22.2	
1990–2004	33200 (32.67%)	32.3	34.3	33.4	
2005–2019	33650 (33.11%)	30.4	34	44.3	
Months between diagnosis and treatment					<0.001
< 1 month	33322 (32.79%)	33.5	35.8	28.8	
1 month	32291 (31.77%)	31.8	34.4	30.7	
≥ 2 months	17084 (16.81%)	15.3	21.9	21.1	
Unknown	18929 (18.63%)	19.4	7.8	19.4	
Status					<0.001
Alive	94228 (92.72%)	6.2	12.9	9.5	
Dead	7398 (7.28%)	93.8	87.1	90.5	

N: number. (a) American Indian/AK Native, Asian/Pacific Islander. (b) Well- and moderately-differentiated cancers. (c) Poorly-differentiated, undifferentiated, and anaplastic cancers. (d) Other primary sites included C34.8 (overlapping lung lesions) and C34.9 (lung, not otherwise specified). (e) Bilateral single primaries and unpaired sites, single unspecified side, and paired sites without information about laterality. (f) Married individuals and those with a domestic partner. (g) Divorced, separated, single (never married), and widowed individuals.

### 3.2 Univariate and stratified analyses

The univariate analysis showed that LS-SPM patients had significantly longer survival durations than patients with LUSC only or combined with other tumors (OT-LUSC). The hazard ratio (HR) for overall survival (OS) in LS-SPM patients was 0.40 (95% confidence interval (CI): 0.39–0.42), which was 0.60 lower than LUSC-only patients (p < 0.05). The HR for OS in OT-LUSC patients was 0.86 (95% CI: 0.84–0.87), a 0.14 decrease compared to LUSC-only patients (p < 0.05). The key covariates included race, sex, grade, primary site, age, historic stage, positive regional nodes, laterality, marital status, radiation, surgery, chemotherapy, year of diagnosis, and duration in months between diagnosis and treatment (**Table 2**). We also performed a stratified analysis and found that the survival duration for LS-SPM patients was considerably longer than that for LUSC-only and OT-LUSC patients (**Supplementary Table S1**).

**Table 2:** Univariate Cox proportional hazards analysis.

Subgroup	Univariate analysis HR (95%CI)	P-value
Sequence number		
Single primary	Reference (1)	
1^st^ of two or more primaries	0.40 (0.39, 0.42)	<0.01
2^nd^ of two or more primaries	0.86 (0.84, 0.87)	<0.01
Sex		
Male	Reference (1)	
Female	0.87 (0.86, 0.89)	<0.01
Age		
< 75 years	Reference (1)	
≥ 75 years	1.33 (1.31, 1.35)	<0.01
Race		
White	Reference (1)	
Black	1.08 (1.05, 1.10)	<0.01
Others^(a)^	1.00 (0.98, 1.03)	0.92
Grade		
Grade 1^(b)^	Reference (1)	
Grade 2^(c)^	1.14 (1.12, 1.16)	<0.01
Unknown	1.49 (1.46, 1.51)	<0.01
Primary site		
Main bronchus	Reference (1)	
Lung lobe	0.67 (0.65, 0.69)	<0.01
Others^(d)^	1.11 (1.08, 1.15)	<0.01
Historic stage		
Localized	Reference (1)	
Regional	1.51 (1.48, 1.54)	<0.01
Distant	3.34 (3.26, 3.41)	<0.01
Unknown	1.85 (1.81, 1.88)	<0.01
Regional nodes		
Not examined	Reference (1)	
Negative	0.38 (0.37, 0.39)	<0.01
Positive	0.65 (0.64, 0.66)	<0.01
Laterality		
Right	Reference (1)	
Left	0.95 (0.94, 0.97)	<0.01
Others^(e)^	1.78 (1.72, 1.85)	<0.01
Marital status		
Partnered^(f)^	Reference (1)	
Alone^(g)^	1.13 (1.11, 1.14)	<0.01
Unknown	1.10 (1.06, 1.14)	<0.01
Radiation		
Yes	Reference (1)	
No	1.37 (1.29, 1.46)	<0.01
Unknown	0.74 (0.73, 0.75)	<0.01
Surgery		
Yes	Reference (1)	
No	3.05 (3.00, 3.10)	<0.01
Unknown	3.42 (3.31, 3.53)	<0.01
Chemotherapy		
Yes	Reference (1)	
No	1.06 (1.05, 1.08)	<0.01
Year of diagnosis		
1975–1989	Reference (1)	
1990–2004	0.93 (0.92, 0.95)	<0.01
2005–2019	0.79 (0.78, 0.81)	<0.01
Months between diagnosis and treatment		
< 1 month	Reference (1)	
1 month	0.97 (0.96, 0.99)	<0.01
> 2 months	0.79 (0.78, 0.81)	<0.01
Unknown	2.31 (2.27, 2.35)	<0.01

(a) American Indian/AK Native, Asian/Pacific Islander. (b) Well- and moderately-differentiated cancers. (c) Poorly-differentiated, undifferentiated, and anaplastic cancers. (d) Other primary sites included C34.8 (overlapping lung lesions) and C34.9 (lung, not otherwise specified). (e) Bilateral single primaries and unpaired sites, single unspecified side, and paired sites without information about laterality. (f) Married individuals and those with a domestic partner. (g) Divorced, separated, single (never married), and widowed individuals.

### 3.3 Multiple regression analysis and Kaplan-Meier curves

**Figure 2:**
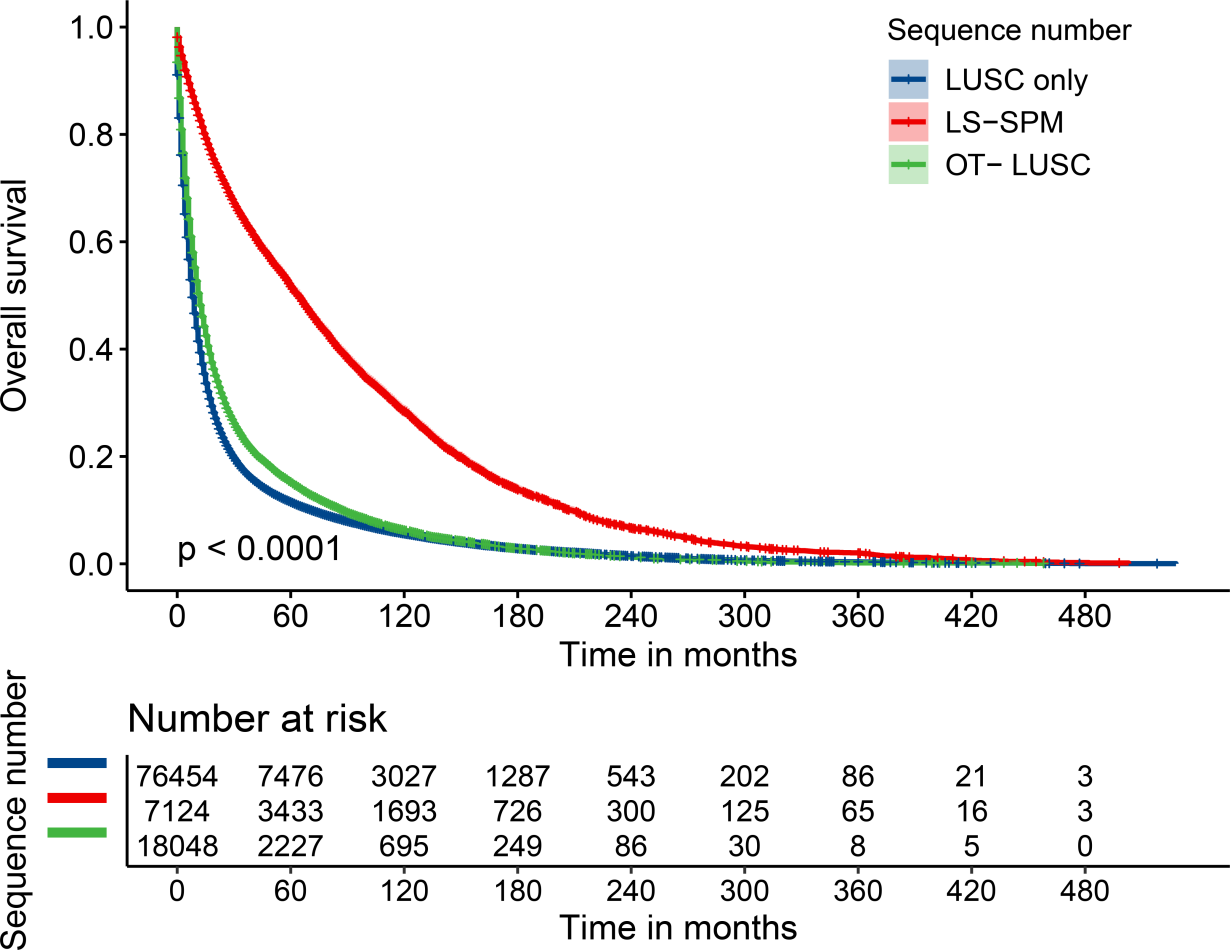
Kaplan-Meier survival curves for LUSCs.

**Figure 3:**
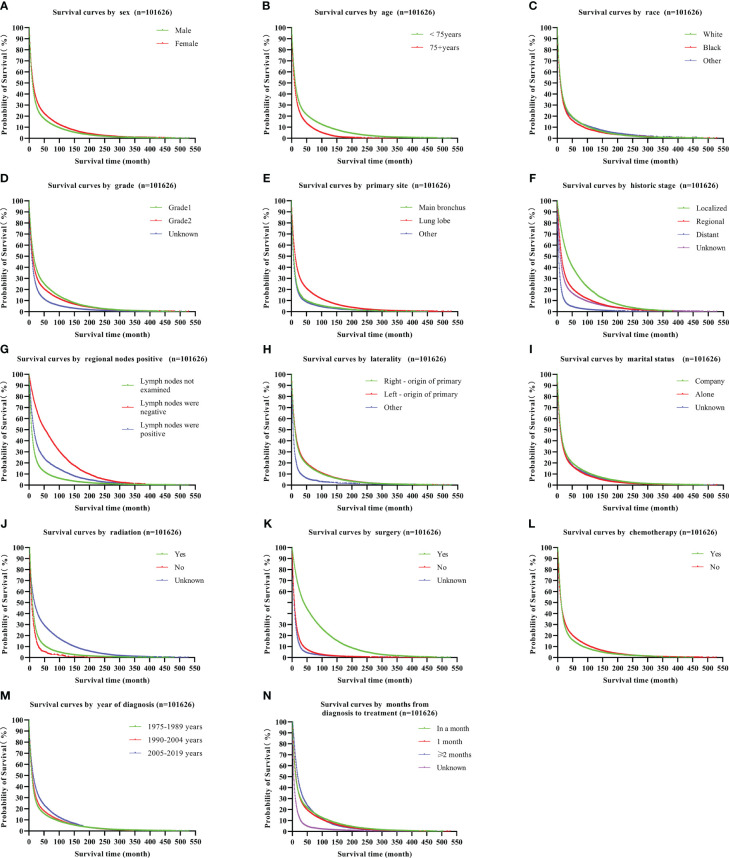
Kaplan-Meier survival curves for LUSC covariates, including sex **(A)**, age **(B)**, race **(C)**, grade **(D)**, primary site **(E)**, historic stage **(F)**, positive regional nodes **(G)**, laterality **(H)**, marital status **(I)**, radiation **(J)**, surgery **(K)**, chemotherapy **(L)**, year of diagnosis **(M)**, and duration in months between diagnosis and treatment **(N)**. LUSC, lung squamous cell carcinoma.

Multiple regression analysis revealed that LS-SPM patients had considerably longer survival periods than other clusters. The unadjusted Model I showed that the HR for OS in LS-SPM patients was 0.40 (95% CI: 0.39–0.42), which was 0.60 lower than that for LUSC-only patients and 0.46 lower than that for OT- LUSC patients. In Model II, adjusted for age, race, and sex, the HR for OS in LS-SPM patients was 0.41 (95% CI: 0.40–0.42), which was 0.59 lower than that for LUSC-only patients and 0.43 lower than that for OT-LUSC patients. Model III was adjusted for sex, race, age, marital status, positive regional nodes, laterality, grade, surgery, summary stage, radiotherapy, primary site, chemotherapy, duration in months between diagnosis and treatment, and year of diagnosis. The HR for OS in LS-SPM patients was 0.54 (95% CI: 0.53–0.56), which was 0.46 lower than that for LUSC-only patients and 0.41 lower than that for OT- LUSC patients. The exposure, time, and outcome variables for this analysis were the sequence number, survival time, and state, respectively. The results were consistent with those of the unadjusted analysis (**Table 3**). We created a Kaplan-Meier survival curve for OS to further assess the survival profile of LS-SPM patients. LS-SPM patients had a slightly superior survival time than LUSC-only and OT-LUSC patients (**Figure 2**). The Kaplan-Meier survival curves for each covariate are shown in **Figure 3**.

**Table 3:** Multiple regression analysis.

Outcome	Model I HR (95%CI); p-value	Model II HR (95%CI); p-value	Model III HR (95%CI); p-value
Sequence number			
Single primary	Reference (1)	Reference (1)	Reference (1)
1^st^ of two or more primaries	0.40 (0.39, 0.42); <0.01	0.41 (0.40, 0.42); <0.01	0.54 (0.53, 0.56); <0.01
2^nd^ of two or more primaries	0.86 (0.84, 0.87); <0.01	0.84 (0.83, 0.86); <0.01	0.95 (0.93, 0.96); <0.01

Model I was unadjusted. Model II was adjusted for age, race, and sex. Model III was adjusted for age, race, sex, regional nodes, laterality, marital status, primary site, grade, summary stage, radiotherapy, surgery, chemotherapy, duration in months between diagnosis and treatment, and year of diagnosis.

### 3.4 Survival time advantage and life table in LS-SPM patients

The median and mean survival times for the 101,626 LUSC patients was 10 (95% CI: 3.88–10.12) months and 32.01 (95% CI: 31.62–32.40) months, respectively. The median and mean survival times for LS-SPM patients were 64 (95% CI: 61.54–66.46) months and 89.11 (95% CI: 86.91–91.30) months, respectively. The median and mean survival times for patients with LUSC alone were 8 (95% CI: 7.89–8.11) months and 26.63 (95% CI: 26.22–27.04) months, respectively. The median and mean survival times for OT-LUSC patients were 12 (95% CI: 11.66–12.34) months and 31.27 (95% CI: 30.42–32.12) months, respectively. The survival times associated with other covariates are shown in **Table 4**. The OS rates for LUSC patients were 21%, 15%, and 7% at 3-, 5- and 10-year, respectively. The survival rates for LS-SPM patients were 62%, 51%, and 28% at 3, 5- and 10-year, respectively. **Table 5** shows the survival times related to the remaining factors.

**Table 4:** Median and mean survival times for LUSC patients.

Groups	Median survival time (months)	Standard Error	95% CI		Mean survival time (months)	Standard Error	95% CI		
			Lower	Upper			Lower	Upper	
**Total**	10.00	0.06	9.88	10.12	32.01	0.20	31.62	32.40	
Sex									
Male	9.00	0.07	8.87	9.13	29.82	0.22	29.38	30.25	
Female	11.00	0.13	10.74	11.26	37.19	0.41	36.38	38.00	
Age									
< 75 years	11.00	0.08	10.85	11.15	36.66	0.26	36.14	37.18	
≥ 75 years	8.00	0.09	7.83	8.17	20.52	0.20	20.12	20.92	
Race									
White	10.00	0.07	9.87	10.13	32.15	0.21	31.74	32.57	
Black	9.00	0.19	8.62	9.37	29.28	0.74	27.83	30.73	
Others^(a)^	9.00	0.24	8.54	9.46	34.51	0.99	32.58	36.44	
Grade									
Grade 1^(b)^	14.00	0.17	13.67	14.33	40.68	0.42	39.86	41.51	
Grade 2^(c)^	10.00	0.10	9.81	10.19	34.77	0.34	34.11	35.44	
Unknown	8.00	0.07	7.85	8.15	21.29	0.26	20.78	21.81	
Sequence number									
Single primary	8.00	0.06	7.89	8.11	26.63	0.21	26.22	27.04	
1^st^ of two or more primaries	64.00	1.25	61.54	66.46	89.11	1.12	86.91	91.30	
2^nd^ of two or more primaries	12.00	0.17	11.66	12.34	31.27	0.44	30.42	32.12	
Primary site									
Main bronchus	6.00	0.14	5.72	6.28	19.26	0.61	18.08	20.45	
Lung lobe	11.00	0.08	10.85	11.15	35.40	0.23	34.95	35.86	
Others^(d)^	5.00	0.09	4.83	5.17	16.47	0.37	15.74	17.20	
Historic stage									
Localized	33.00	0.52	31.98	34.02	61.86	0.64	60.60	63.11	
Regional	14.00	0.15	13.71	14.29	36.63	0.43	35.79	37.47	
Distant	4.00	0.05	3.90	4.10	11.02	0.20	10.63	11.41	
Unknown	9.00	0.08	8.84	9.16	29.76	0.31	29.15	30.37	
Regional nodes									
Not examined	8.00	0.05	7.90	8.10	22.56	0.18	22.22	22.91	
Negative	51.00	0.95	49.13	52.87	78.66	0.79	77.11	80.21	
Positive	15.00	0.27	14.47	15.54	42.02	0.75	40.54	43.49	
Laterality									
Right	10.00	0.08	9.84	10.16	31.27	0.27	31.27	32.33	
Left	11.00	0.10	10.80	11.20	33.10	0.31	33.10	34.32	
Others^(e)^	4.00	0.14	3.73	4.27	11.93	0.71	11.93	14.72	
Marital status									
Partnered^(f)^	11.00	0.09	10.83	11.17	34.49	0.27	33.96	35.03	
Alone^(g)^	9.00	0.09	8.82	9.18	28.32	0.30	27.74	28.90	
Unknown	9.00	0.33	8.36	9.64	30.75	1.19	28.41	33.08	
Radiation recoded									
Yes	9.00	0.06	8.88	9.12	22.22	0.20	21.83	22.61	
No	6.00	0.35	5.32	6.68	13.01	0.85	11.33	14.68	
Unknown	11.00	0.15	10.72	11.29	42.92	0.35	42.23	43.61	
Surgery recoded									
Yes	37.00	0.47	36.08	37.92	70.16	0.52	69.15	71.18	
No	7.00	0.05	6.91	7.09	15.58	0.14	15.30	15.86	
Unknown	6.00	0.14	5.73	6.27	12.72	0.42	11.91	13.54	
Chemotherapy recoded									
Yes	11.00	0.11	10.79	11.21	31.09	0.47	30.17	32.01	
No	9.00	0.07	8.86	9.14	32.33	0.23	31.89	32.78	
Year of diagnosis									
1975–1989	8.00	0.08	7.84	8.16	27.95	0.30	27.36	28.54	
1990–2004	10.00	0.10	9.80	10.20	30.24	0.30	29.66	30.83	
2005–2019	13.00	0.14	12.72	13.28	33.98	0.30	33.39	34.57	
Months between diagnosis and treatment									
< 1 month	10.00	0.11	9.78	10.22	36.50	0.38	35.76	37.23	
1 month	12.00	0.12	11.77	12.23	35.17	0.37	34.45	35.88	
≥ 2 months	18.00	0.22	17.57	18.43	40.03	0.50	39.05	41.01	
Unknown	2.00	0.05	1.91	2.10	9.89	0.22	9.45	10.33	

(a) American Indian/AK Native, Asian/Pacific Islander. (b) Well- and moderately-differentiated cancers. (c) Poorly-differentiated, undifferentiated, and anaplastic cancers. (d) Other primary sites included C34.8 (overlapping lung lesions) and C34.9 (lung, not otherwise specified). (e) Bilateral single primaries and unpaired sites, single unspecified side, and paired sites without information about laterality. (f) Married individuals and those with a domestic partner. (g) Divorced, separated, single (never married), and widowed individuals.

**Table 5:** The 3-year and 5-year survival rates for LUSC patients.

Groups	Percentage of total patients (%)	3-year survival rate (%)	Probability density	5-year survival rate (%)	Probability density	10-year survival rate (%)	Probability density
**Total**	100.00	0.21	< 0.01	0.15	< 0.01	0.07	< 0.01
Sex							
Male	69.76	0.19	< 0.01	0.13	< 0.01	0.07	< 0.01
Female	30.24	0.24	< 0.01	0.18	< 0.01	0.09	< 0.01
Age							
< 75 years	69.81	0.23	< 0.01	0.17	< 0.01	0.09	< 0.01
≥ 75 years	30.19	0.15	< 0.01	0.09	< 0.01	0.03	< 0.01
Race							
White	85.31	0.21	< 0.01	0.15	< 0.01	0.07	< 0.01
Black	8.22	0.18	< 0.01	0.12	< 0.01	0.06	< 0.01
Others^(a)^	6.47	0.20	< 0.01	0.15	< 0.01	0.08	< 0.01
Grade							
Grade 1^(b)^	27.03	0.27	< 0.01	0.20	< 0.01	0.10	< 0.01
Grade 2^(c)^	37.42	0.22	< 0.01	0.16	< 0.01	0.08	< 0.01
Unknown	35.54	0.13	< 0.01	0.08	< 0.01	0.04	< 0.01
Sequence number							
Single primary	75.23	0.16	< 0.01	0.11	< 0.01	0.05	< 0.01
1^st^ of two or more primaries	7.01	0.62	< 0.01	0.51	< 0.01	0.28	< 0.01
2^nd^ of two or more primaries	17.76	0.22	< 0.01	0.15	< 0.01	0.06	< 0.01
Primary site							
Main bronchus	5.96	0.11	< 0.01	0.07	< 0.01	0.04	< 0.01
Lung lobe	81.28	0.23	< 0.01	0.17	< 0.01	0.08	< 0.01
Other^(d)^	12.75	0.09	< 0.01	0.06	< 0.01	0.03	< 0.01
Historic stage							
Localized	14.33	0.46	< 0.01	0.34	< 0.01	0.17	< 0.01
Regional	22.00	0.24	< 0.01	0.17	< 0.01	0.08	< 0.01
Distant	21.80	0.05	< 0.01	0.03	< 0.01	0.01	< 0.01
Unknown	41.88	0.18	< 0.01	0.13	< 0.01	0.07	< 0.01
Regional nodes							
Not examined	76.86	0.14	< 0.01	0.09	< 0.01	0.04	< 0.01
Negative	13.40	0.56	< 0.01	0.45	< 0.01	0.24	< 0.01
Positive	9.74	0.27	< 0.01	0.20	< 0.01	0.11	< 0.01
Laterality							
Right	53.98	0.21	< 0.01	0.15	< 0.01	0.07	< 0.01
Left	42.69	0.22	< 0.01	0.16	< 0.01	0.08	< 0.01
Others^(e)^	3.32	0.06	< 0.01	0.04	< 0.01	0.02	< 0.01
Marital status							
Partnered^(f)^	57.61	0.22	< 0.01	0.16	< 0.01	0.08	< 0.01
Alone^(g)^	39.15	0.19	< 0.01	0.13	< 0.01	0.06	< 0.01
Unknown	3.25	0.19	< 0.01	0.13	< 0.01	0.07	< 0.01
Radiation recoded							
Yes	52.51	0.13	< 0.01	0.08	< 0.01	0.03	< 0.01
No	0.95	0.06	< 0.01	0.03	< 0.01	0.01	< 0.01
Unknown	46.54	0.29	< 0.01	0.22	< 0.01	0.12	< 0.01
Surgery recoded							
Yes	29.17	0.49	< 0.01	0.38	< 0.01	0.20	< 0.01
No	66.37	0.09	< 0.01	0.05	< 0.01	0.02	< 0.01
Unknown	4.46	0.05	< 0.01	0.03	< 0.01	0.01	< 0.01
Chemotherapy recoded							
Yes	25.41	0.19	< 0.01	0.13	< 0.01	0.06	< 0.01
No	74.59	0.21	< 0.01	0.15	< 0.01	0.08	< 0.01
Year of diagnosis							
1975–1989	34.22	0.17	< 0.01	0.12	< 0.01	0.06	< 0.01
1990–2004	32.67	0.19	< 0.01	0.14	< 0.01	0.07	< 0.01
2005–2019	33.11	0.26	< 0.01	0.18	< 0.01	0.09	< 0.01
Months between diagnosis and treatment							
< 1 month	32.79	0.23	< 0.01	0.17	< 0.01	0.09	< 0.01
1 month	31.77	0.23	< 0.01	0.16	< 0.01	0.08	< 0.01
≥ 2 months	16.81	0.29	< 0.01	0.20	< 0.01	0.08	< 0.01
Unknown	18.63	0.05	< 0.01	0.02	< 0.01	0.01	< 0.01

(a) American Indian/AK Native, Asian/Pacific Islander. (b) Well- and moderately-differentiated cancers. (c) Poorly-differentiated, undifferentiated, and anaplastic cancers. (d) Other primary sites included C34.8 (overlapping lung lesions) and C34.9 (lung, not otherwise specified). (e) Bilateral single primaries and unpaired sites, single unspecified side, and paired sites without information about laterality. (f) Married individuals and those with a domestic partner. (g) Divorced, separated, single (never married), and widowed individuals.

## 4 Discussion

With the incidence of LUSC increasing year by year, the number of LUSC patients with other cancers has also risen significantly (6). However, there have been relatively few studies on LUSC combined with SPM in clinical practice, and the availability of relevant data and information is limited (17). Hence, there is an urgent need for additional exploratory studies to conduct prognostic analysis on patients with LUSC combined with SPM.

In this study, we investigated the prognostic impact of combined SPM by analyzing data from LUSC-only patients in the SEER database. We employed such as univariate, multiple regression, and survival analyses to provide robust scientific support for prognostic assessment. We plotted the Kaplan-Meier survival curves for comparison using GraphPad Prism 9 software, which provided a clearer and more intuitive representation of the study results. The study’s findings revealed a significant difference in the survival rates of patients with LUSC and SPM. Specifically, LS-SPM patients exhibited better survival and prognosis compared to LUSC-only patients. Through a meticulous comparison with prior research, this study comprehensively considered potential confounding factors, including age, gender, stage, and treatment regimen, while analyzing survival rate discrepancies. This comprehensive analysis enabled a more thorough understanding of the impact of combined tumors on prognosis, thereby offering clinicians a more precise reference for decision-making.

The occurrence of SPMs may also be related to the first primary type, genetic susceptibility, cancer susceptibility syndrome, and the treatment administered for the first primary (18). Multiple factors contribute to the development of SPM in patients with squamous lung cancer, such as the course of lung cancer treatment, smoking, genetic factors, environmental factors, and the immune system (19). There is evidence that the incidence of multiple primary malignant tumors in the same or different organ systems ranges from 2% to 17% (12, 20–22). The development of second primary tumors is a multifactorial process, not just a random event. Understanding the relationship between these factors is critical to preventing and managing the risk of second primary tumors. Compared to metastatic and recurrent malignancies, early diagnosis and aggressive therapy in multiple primary cancers have a greater impact on survival and prognosis (23). In addition, the present study demonstrated a statistically significant difference between OT-LUSC patients and those with LUSC alone. The survival rate for OT-LUSC patients was lower than that for LS-SPM patients. This suggests that the survival and prognosis depend greatly on the sequence of LUSC progression. Our study offers an indication of the prognosis for patients and their families, and provides a reference for individualized treatment and follow-up plans in LUSCs.

Previous studies have also performed covariate analyses in a manner similar to the present study. Increasing age causes gradual deterioration of body functions, and is often associated with various geriatric syndromes and chronic diseases, which results in a considerable decline in treatment tolerance (24, 25). Therefore, the prognosis and quality of life in elderly patients are unsatisfactory. Studies have shown that 90% of the lung cancer diagnoses and fatalities occur in individuals aged > 55 years in the USA, where the average age at diagnosis is 71 years (26). This may explain why LUSC patients aged ≥ 75 years have worse median survival durations in the USA. A few studies have reported a poorer prognosis for malignancy in the main bronchus compared to those in other locations (27). However, in the present study, LS-SPM patients had a higher primary site survival rate in the main bronchus than in lung lobes. This was significantly different compared to previous studies, and provides a different survival concept to determine the prognosis of LS-SPMs based on the primary site. Osarogiagbon et al. (28) reported that identification of lymph node metastases is critical for the selection of appropriate postoperative adjuvant therapy. Urban et al. (29) suggested that lymph node involvement was an important prognostic factor in surgical resection cases, and provided an indication for adjuvant chemotherapy. Lymph node staging is critical in determining treatment strategies and prognosis for patients with lung cancer (30). Our study showed that patients with LUSC who had negative lymph node tests had a longer median survival time compared to patients with positive results. Although the percentage of patients who did not receive lymph node testing in the data was 76.86%, the number of patients who received lymph node testing was 23,521. Compared with other studies, our study was more rigorous and less biased. Moreover, the focus of the study was on the comparison of prognosis between positive or negative results of lymph node testing, and patients who did not receive lymph node testing were not the main target of our study. Furthermore, our study found that female SPM patients had better OS rates compared to males. The higher immunological response in females may provide a survival advantage. Zandman-Goddard et al. (31) suggested that enhanced immunity is associated with estrogen-regulated immune function in females.

Moreover, this study’s comparison of survival rates and influencing factors sheds light on the association between different primary tumors and squamous lung cancer, introducing novel insights into the mechanism of tumor coexistence. It deepens our comprehension of inter-tumor relationships, fostering fresh ideas and avenues for investigating disease mechanisms, and positively impacting the field of tumor research. These findings hold remarkable significance for prognostic assessment and clinical management of LUSC patients with concurrent tumors. Beyond averting haphazard treatments and optimizing patient care plans, the research aids in curbing unnecessary medical expenses and mitigating drug side effects, thereby enhancing patients’ quality of life. Concurrently, the prognostic insights gleaned here offer invaluable guidance for long-term patient monitoring and follow-up, facilitating timely treatment adjustments. Furthermore, the outcomes of this study can serve as a foundation for further in-depth research in related fields, propelling advancements in mechanistic understanding, drug development, and treatment strategy innovation.

The present study also had some limitations. This was a retrospective study and further studies are required to corroborate these findings. Studies with additional LUSC data from other nations may provide more persuasive findings. In addition, history of smoking and alcohol use, and body mass index were not included in the SEER database. It may be useful to investigate these factors to better characterize survival in SPM.

## 5 Conclusions

The prognosis and survival of LS-SPM was better than that for LUSC-only and OT-LUSC patients. SPMs are not always a sign of poor prognosis in LUSC patients. Active treatment and immune monitoring should be provided to LS-SPM patients. The present study may assist policy makers in monitoring public health measures and implementing policies to lower the mortality rates in LS-SPM patients.

## Definitions and Abbreviations

LUSC, lung squamous-cell carcinoma; SPM, second primary malignancy; LS-SPM, LUSC patients secondary to other primary malignancies (1^st^ of two or more primaries); OT-LUSC, other primary cancer secondary to LUSC (2^nd^ of two or more primaries); Age < 1 year old: age; Sequence number, defines the quantity and timing of all detectable primary tumors, including borderline, benign, and *in situ* malignant tumors; Vital status recode, status; Reason no cancer-directed surgery, surgery; Race recode (White, Black, Other): race; Summary stage: Historic stage; Chemotherapy recode (yes, no/unknown): chemotherapy; Marital status at diagnosis: marital status; Grade (through 2017): grade; Radiation recode, radiation; Regional nodes positive (1988+), regional nodes positive; Primary Site–labelled, Primary Site.

## Data availability statement

The datasets presented in this study can be found in online repositories. The names of the repository/repositories and accession number(s) can be found below: https://seer.cancer.gov/.

## Ethics statement

Ethical approval was not required for the studies involving humans because The National Center for Health Statistics’ Ethical Review Committee authorized the SEER procedures. After obtaining a research license (serial number: 14027-Nov2021), we used SEER*Stat version 8.4.0.1 to retrieve the SEER Research Plus Data (covering eight registries; 1975–2019) released in November 2021. Informed consent was not required because patient data were obtained from public databases. The studies were conducted in accordance with the local legislation and institutional requirements. Written informed consent for participation was not required from the participants or the participants’ legal guardians/next of kin in accordance with the national legislation and institutional requirements because The National Center for Health Statistics’ Ethical Review Committee authorized the SEER procedures.

## Author contributions

WH: Data curation, Formal analysis, Methodology, Supervision, Writing – original draft, Writing – review & editing. SW: Data curation, Methodology, Writing – original draft, Writing – review & editing. YW: Data curation, Formal analysis, Funding acquisition, Supervision, Investigation, Resources, Writing – review & editing. LS: Investigation, Conceptualization, Writing – review & editing. JX: Conceptualization, Formal analysis, Funding acquisition, Investigation, Writing – review & editing.
