# Supplementary material for: Prognostic analysis of lung squamous cell carcinoma patients with second primary malignancies: a SEER database study
Source: Front Oncol. 2024 Feb 20;14:1294383. doi: 10.3389/fonc.2024.1294383 (PMC10912175; doi:10.3389/fonc.2024.1294383)
Supplement: Supplementary file 1 [file Table_1.docx]

**Table S1. Stratified analysis of LUSC patients.**

| **Subgroup** | **N** | **Status** | **P-value** |
| --- | --- | --- | --- |
|  |  | **HR (95% CI)** |  |
| **Sex** |  |  |  |
| **Male** |  |  |  |
| Single primary | 54177 | Reference (1) |  |
| 1^st^ of two or more primaries | 5027 | 0.40 (0.39, 0.42) | <0.01 |
| 2^nd^ of two or more primaries | 11688 | 0.89 (0.87, 0.91) | <0.01 |
| **Female** |  |  |  |
| Single primary | 22277 | Reference (1) |  |
| 1^st^ of two or more primaries | 2097 | 0.40 (0.38, 0.42) | <0.01 |
| 2^nd^ of two or more primaries | 6360 | 0.82 (0.80, 0.85) | <0.01 |
| **Age** |  |  |  |
| **< 75 years** |  |  |  |
| Single primary | 54633 | Reference (1) |  |
| 1^st^ of two or more primaries | 5575 | 0.40 (0.39, 0.41) | <0.01 |
| 2^nd^ of two or more primaries | 10734 | 0.82 (0.80, 0.84) | <0.01 |
| ≥ **75 years** |  |  |  |
| Single primary | 21821 | 1 |  |
| 1^st^ of two or more primaries | 1549 | 0.45 (0.42, 0.47) | <0.01 |
| 2^nd^ of two or more primaries | 7314 | 0.85 (0.83, 0.88) | <0.01 |
| **Race** |  |  |  |
| **White** |  |  |  |
| Single primary | 64776 | Reference (1) |  |
| 1^st^ of two or more primaries | 6132 | 0.41 (0.40, 0.42) | <0.01 |
| 2^nd^ of two or more primaries | 15790 | 0.86 (0.85, 0.88) < | <0.01 |
| **Black** |  |  |  |
| Single primary | 6420 | 1 |  |
| 1^st^ of two or more primaries | 584 | 0.38 (0.35, 0.42) | <0.01 |
| 2^nd^ of two or more primaries | 1348 | 0.85 (0.80, 0.90) | <0.01 |
| **Others^(a)^** |  |  |  |
| Single primary | 5258 | Reference (1) |  |
| 1^st^ of two or more primaries | 408 | 0.40 (0.36, 0.45) | <0.01 |
| 2^nd^ of two or more primaries | 910 | 0.83 (0.77, 0.90) | <0.01 |
| **Grade** |  |  |  |
| **Grade 1^(b)^** |  |  |  |
| Single primary | 19730 | Reference (1) |  |
| 1^st^ of two or more primaries | 2587 | 0.43 (0.41, 0.45) | <0.01 |
| 2^nd^ of two or more primaries | 5156 | 0.89 (0.86, 0.92) | <0.01 |
| **Grade 2^(c)^** |  |  |  |
| Single primary | 28972 | Reference (1) |  |
| 1^st^ of two or more primaries | 2863 | 0.40 (0.39, 0.42) | <0.01 |
| 2^nd^ of two or more primaries | 6197 | 0.85 (0.82, 0.87) | <0.01 |
| **Unknown** |  |  |  |

**Continued table S1.**

| **Sub-group** | **N** | **Status** | **P - value** |
| --- | --- | --- | --- |
|  |  | **HR (95% CI)** |  |
| Single primary | 27752 | Reference (1) |  |
| 1^st^ of two or more primaries | 1674 | 0.42 (0.40, 0.44) | <0.01 |
| 2^nd^ of two or more primaries | 6695 | 0.85 (0.83, 0.88) | <0.01 |
| **Primary site** |  |  |  |
| **Main bronchus** |  |  |  |
| Single primary | 4994 | Reference (1) |  |
| 1^st^ of two or more primaries | 256 | 0.42 (0.37, 0.48) | <0.01 |
| 2^nd^ of two or more primaries | 810 | 0.92 (0.86, 1.00) | 0.04 |
| **Lung lobe** |  |  |  |
| Single primary | 60892 | Reference (1) |  |
| 1^st^ of two or more primaries | 6426 | 0.42 (0.40, 0.43) | <0.01 |
| 2^nd^ of two or more primaries | 15287 | 0.87 (0.85, 0.89) | <0.01 |
| **Others^(d)^** |  |  |  |
| Single primary | 10568 | Reference (1) |  |
| 1^st^ of two or more primaries | 442 | 0.39 (0.36, 0.44) | <0.01 |
| 2^nd^ of two or more primaries | 1951 | 0.89 (0.85, 0.93) | <0.01 |
| **Historic stage** |  |  |  |
| **Localized** |  |  |  |
| Single primary | 8562 | Reference (1) |  |
| 1^st^ of two or more primaries | 2155 | 0.56 (0.53, 0.59) | <0.01 |
| 2^nd^ of two or more primaries | 3847 | 1.03 (0.99, 1.08) | 0.11 |
| **Regional** |  |  |  |
| Single primary | 16348 | Reference (1) |  |
| 1^st^ of two or more primaries | 1794 | 0.43 (0.41, 0.45) | <0.01 |
| 2^nd^ of two or more primaries | 4211 | 0.96 (0.93, 0.99) | 0.02 |
| **Distant** |  |  |  |
| Single primary | 17828 | Reference (1) |  |
| 1^st^ of two or more primaries | 560 | 0.43 (0.40, 0.47) | <0.01 |
| 2^nd^ of two or more primaries | 3762 | 0.93 (0.90, 0.97) | <0.01 |
| **Unknown** |  |  |  |
| Single primary | 33716 | Reference (1) |  |
| 1^st^ of two or more primaries | 2615 | 0.41 (0.40, 0.43) | <0.01 |
| 2^nd^ of two or more primaries | 6228 | 0.86 (0.84, 0.89) | <0.01 |
| **Regional nodes** |  |  |  |
| **Not examined** |  |  |  |
| Single primary | 60861 | Reference (1) |  |
| 1^st^ of two or more primaries | 3943 | 0.41 (0.39, 0.42) | <0.01 |
| 2^nd^ of two or more primaries | 13301 | 0.88 (0.86, 0.90) | <0.01 |
| **Negative** |  |  |  |
| Single primary | 8240 | Reference (1) |  |
| 1^st^ of two or more primaries | 2356 | 0.63 (0.60, 0.66) | <0.01 |

**Continued table S1.**

| **Sub-group** | **N** | **Status** | **P - value** |
| --- | --- | --- | --- |
|  |  | **HR (95% CI)** |  |
| 2^nd^ of two or more primaries | 3026 | 1.03 (0.99, 1.08) | 0.16 |
| **Positive** |  |  |  |
| Single primary | 7353 | 1 |  |
| 1^st^ of two or more primaries | 825 | 0.45 (0.42, 0.49) | <0.01 |
| 2^nd^ of two or more primaries | 1721 | 0.97 (0.92, 1.03) | 0.36 |
| **Laterality** |  |  |  |
| **Right** |  |  |  |
| Single primary | 41386 | Reference (1) |  |
| 1^st^ of two or more primaries | 3732 | 0.41 (0.40, 0.42) | <0.01 |
| 2^nd^ of two or more primaries | 9743 | 0.86 (0.84, 0.88) | <0.01 |
| **Left** |  |  |  |
| Single primary | 32252 | Reference (1) | 1 |
| 1^st^ of two or more primaries | 3311 | 0.40 (0.39, 0.42) | <0.01 |
| 2^nd^ of two or more primaries | 7825 | 0.86 (0.84, 0.89) | <0.01 |
| **Others^(e)^** |  |  |  |
| Single primary | 2816 | 1 |  |
| 1^st^ of two or more primaries | 81 | 0.44 (0.35, 0.56) | <0.01 |
| 2^nd^ of two or more primaries | 480 | 0.91 (0.82, 1.00) | 0.05 |
| **Marital status** |  |  |  |
| **Partnered^(f)^** |  |  |  |
| Single primary | 44022 | Reference (1) |  |
| 1^st^ of two or more primaries | 4417 | 0.40 (0.39, 0.42) | <0.01 |
| 2^nd^ of two or more primaries | 10103 | 0.88 (0.86, 0.90) | <0.01 |
| **Alone^(g)^** |  |  |  |
| Single primary | 30018 | Reference (1) |  |
| 1^st^ of two or more primaries | 2518 | 0.41 (0.39, 0.43) | <0.01 |
| 2^nd^ of two or more primaries | 7246 | 0.82 (0.80, 0.85) | <0.01 |
| **Unknown** |  |  |  |
| Single primary | 2414 | Reference (1) |  |
| 1^st^ of two or more primaries | 189 | 0.44 (0.37, 0.51) | <0.01 |
| 2^nd^ of two or more primaries | 699 | 0.81 (0.74, 0.89) | <0.01 |
| **Radiation** |  |  |  |
| **Yes** |  |  |  |
| Single primary | 42210 | 1 |  |
| 1^st^ of two or more primaries | 2543 | 0.40 (0.39, 0.42) | <0.01 |
| 2^nd^ of two or more primaries | 8606 | 0.85 (0.83, 0.87) | <0.01 |
| **No** |  |  |  |
| Single primary | 775 | Reference (1) |  |
| 1^st^ of two or more primaries | 37 | 0.53 (0.37, 0.75) | <0.01 |
| 2^nd^ of two or more primaries | 156 | 1.04 (0.88, 1.24) | 0.63 |
| **Unknown** |  |  |  |

**Continued table S1.**

| **Sub-group** | **N** | **Status** | **P - value** |
| --- | --- | --- | --- |
|  |  | **HR (95% CI)** |  |
| Single primary | 33469 | Reference (1) |  |
| 1^st^ of two or more primaries | 4544 | 0.44 (0.42, 0.45) | <0.01 |
| 2^nd^ of two or more primaries | 9286 | 0.90 (0.88, 0.92) | <0.01 |
| **Surgery** |  |  |  |
| **Yes** |  |  |  |
| Single primary | 19626 | Reference (1) |  |
| 1^st^ of two or more primaries | 4533 | 0.55 (0.53, 0.57) | <0.01 |
| 2^nd^ of two or more primaries | 5482 | 0.98 (0.95, 1.02) | 0.32 |
| **No** |  |  |  |
| Single primary | 52933 | Reference (1) |  |
| 1^st^ of two or more primaries | 2477 | 0.46 (0.44, 0.48) | <0.01 |
| 2^nd^ of two or more primaries | 12043 | 0.85 (0.83, 0.86) | <0.01 |
| **Unknown** |  |  |  |
| Single primary | 3895 | Reference (1) |  |
| 1^st^ of two or more primaries | 114 | 0.51 (0.42, 0.62) | <0.01 |
| 2^nd^ of two or more primaries | 523 | 0.87 (0.80, 0.96) | <0.01 |
| **Chemotherapy** |  |  |  |
| **Yes** |  |  |  |
| Single primary | 19760 | Reference (1) |  |
| 1^st^ of two or more primaries | 1548 | 0.40 (0.37, 0.42) | <0.01 |
| 2^nd^ of two or more primaries | 4512 | 0.86 (0.83, 0.89) | <0.01 |
| **No** |  |  |  |
| Single primary | 56694 | Reference (1) |  |
| 1^st^ of two or more primaries | 5576 | 0.41 (0.40, 0.42) | <0.01 |
| 2^nd^ of two or more primaries | 13536 | 0.86 (0.84, 0.87) | <0.01 |
| **Year of diagnosis** |  |  |  |
| **1975–1989** |  |  |  |
| Single primary | 28509 | Reference (1) |  |
| 1^st^ of two or more primaries | 2257 | 0.40 (0.38, 0.42) | <0.01 |
| 2^nd^ of two or more primaries | 4010 | 0.91 (0.88, 0.94) | <0.01 |
| **1990–2004** |  |  |  |
| Single primary | 24721 | Reference (1) |  |
| 1^st^ of two or more primaries | 2442 | 0.40 (0.38, 0.41) | <0.01 |
| 2^nd^ of two or more primaries | 6037 | 0.90 (0.87, 0.92) | <0.01 |
| **2005–2019** |  |  |  |
| Single primary | 23224 | Reference (1) |  |
| 1^st^ of two or more primaries | 2425 | 0.43 (0.41, 0.45) | <0.01 |
| 2^nd^ of two or more primaries | 8001 | 0.86 (0.83, 0.88) | <0.01 |
| **Months between diagnosis and treatment** |  |  |  |
| **< 1 month** |  |  |  |
| Single primary | 25577 | Reference (1) |  |

**Continued table S1.**

| **Sub-group** | **N** | **Status** | **P - value** |
| --- | --- | --- | --- |
|  |  | **HR (95% CI)** |  |
| 1^st^ of two or more primaries | 2552 | 0.39 (0.38, 0.41) | <0.01 |
| 2^nd^ of two or more primaries | 5193 | 0.86 (0.83, 0.88) | <0.01 |
| **1 month** |  |  |  |
| Single primary | 24301 | Reference (1) |  |
| 1^st^ of two or more primaries | 2454 | 0.41 (0.39, 0.42) | <0.01 |
| 2^nd^ of two or more primaries | 5536 | 0.85 (0.82, 0.88) | <0.01 |
| **≥ 2 months** |  |  |  |
| Single primary | 11706 | Reference (1) |  |
| 1^st^ of two or more primaries | 1562 | 0.48 (0.46, 0.51) | <0.01 |
| 2^nd^ of two or more primaries | 3816 | 0.89 (0.85, 0.92) | <0.01 |
| **Unknown** |  |  |  |
| Single primary | 14870 | Reference (1) |  |
| 1^st^ of two or more primaries | 556 | 0.56 (0.52, 0.62) | <0.01 |
| 2^nd^ of two or more primaries | 3503 | 0.89 (0.86, 0.93) | <0.01 |

**Note:** (a) American Indian/AK Native, Asian/Pacific Islander. (b) Well- and moderately-differentiated cancers. (c) Poorly-differentiated, undifferentiated, and anaplastic cancers. (d) Other primary sites included C34.8 (overlapping lung lesions) and C34.9 (lung, not otherwise specified). (e) Bilateral single primaries and unpaired sites, single unspecified side, and paired sites without information about laterality. (f) Married individuals and those with a domestic partner. (g) Divorced, separated, single (never married), and widowed individuals.
